# Cell‐free DNA analysis for recurrent respiratory papillomatosis: A case report

**DOI:** 10.1002/ccr3.9268

**Published:** 2024-08-06

**Authors:** Satoshi Yamada, Kotaro Kano, Ryuji Ishikawa, Atsushi Imai, Daiki Mochizuki, Kotaro Morita, Kazutaka Takeuchi, Yoshinori Takizawa, Hideya Kawasaki, Kiyoshi Misawa

**Affiliations:** ^1^ Department of Otolaryngology/Head and Neck Surgery Hamamatsu University School of Medicine Hamamatsu Japan; ^2^ Department of Otolaryngology Yaizu City Hospital Yaizu Japan; ^3^ Institute for NanoSuit Research, Preeminent Medical Photonics Education and Research Center Hamamatsu University School of Medicine Hamamatsu Japan

**Keywords:** cfDNA, human papillomavirus, recurrent respiratory papillomatosis, viral load

## Abstract

A 35‐year‐old male presented with recurrent respiratory papillomatosis. Human papillomavirus type 11 was detected from all sites of tumor tissue DNA by PCR. The pre‐surgery cell‐free DNA (cfDNA) viral load (3.33 × 10^3^ copies/ng DNA) fell below the post‐surgical detection limits on achieving remission, suggesting cfDNA's potential as a biomarker.

## Graphical abstract

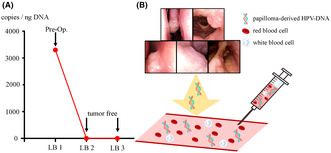

## 1 INTRODUCTION

Recurrent respiratory papillomatosis (RRP) has the potential to disseminate widely throughout the airways, often making disease control difficult.^1^ It is often caused by human papillomavirus (HPV) types 6 or 11.^2^ Persistent infection with HPV viral particles correlates with the disease severity of RRP.^3^ The Derkay Score is a useful assessment method to conveniently determine severity^4^; however, predicting when recurrence may occur or the speed of tumor growth remains a challenge. Understanding the course of the disease could provide crucial information for treatment plans, such as the follow‐up intervals and the necessity for surgery.

In HPV‐associated oropharyngeal cancer, the presence or absence of HPV DNA in cell‐free DNA (cfDNA) in the blood has gained attention as a biomarker to predict recurrence and monitoring the disease course.^5^, ^6^ We hypothesized that analyzing HPV DNA in cfDNA could also be valuable to assess the disease course of RRP.

To our knowledge, this is the first analysis to evaluate the usefulness of cfDNA for patients with RRP. The purpose of this case report is to assess whether tumor‐derived cfDNA analysis, similar to that for HPV‐related oropharyngeal cancer, is feasible in benign tumors like RRP, and if feasible, whether it could serve as a marker to assess disease severity.

## 2 CASE HISTORY/EXAMINATION

**FIGURE 1:**
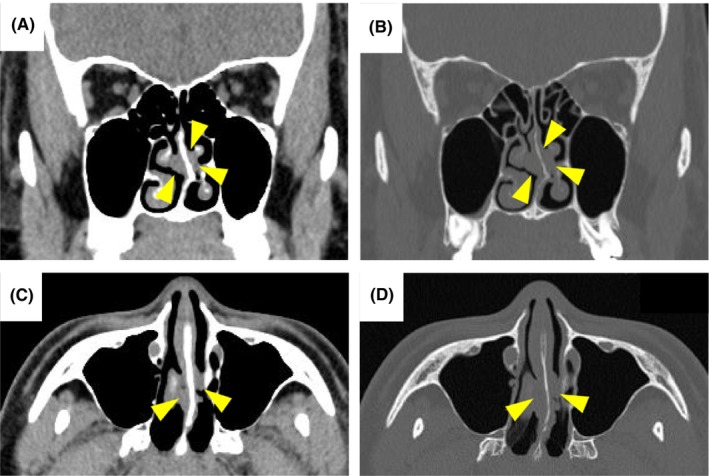
Computed tomography findings of the coronal view (A), coronal view with bone window (B), axial view (C), and axial view with bone window (D) are shown. A mass is visible in the nasal septum (arrowhead). No obscuration of the perpendicular plate of the ethmoid bone is present.

**FIGURE 2:**
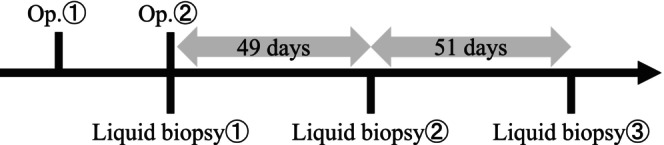
The timeline for this case: (1) A liquid biopsy was performed immediately before surgery and (2) the remaining two liquid biopsies were performed during the outpatient follow‐up. Op, operation.

A 35‐year‐old male presented at our hospital with nasal congestion. The patient had no history of previous illnesses or complications and was not receiving any regular medication. History of alcohol consumption or smoking was also absent. Additionally, the patient had not received the HPV vaccine. A tumor was identified in the nasal septum, obscuring visibility beyond its depth. Computed tomography (CT) scans revealed a mass in the nasal septum but did not show bone obscuration of the perpendicular plate of the ethmoid bone (Figure 1A–D). No obvious neoplastic lesions or signs of chronic rhinosinusitis were observed in other areas. Biopsy confirmed a diagnosis of inverted papilloma. The treatment course and timing of the cfDNA analysis are shown in Figure 2.

**FIGURE 3:**
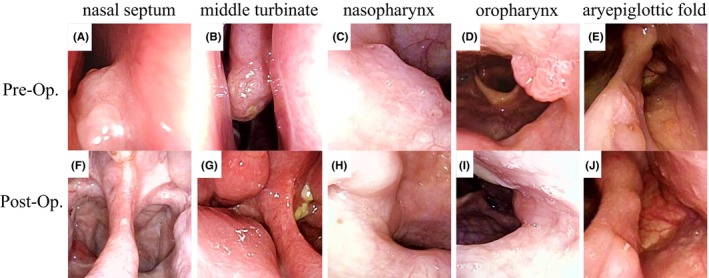
The findings of the nasal septum (A, F), middle turbinate (B, G), nasopharynx (C, H), oropharynx (D, I), and aryepiglottic fold (E, J) before and after the surgery. No signs of recurrence were observed during the follow‐up period. Pre‐Op, preoperative; Post‐Op, postoperative.

**FIGURE 4:**
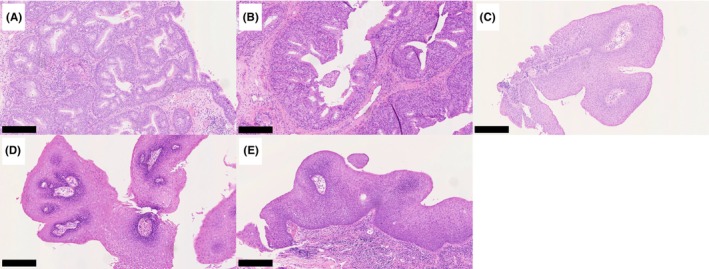
Hematoxylin and eosin (HE) staining of tumors in the nasal septum (A), middle turbinate (B), nasopharynx (C), oropharynx (D), and aryepiglottic fold (E). The nasal septum and middle nasal turbinate tumors showed inverted papilloma, whereas the others presented squamous epithelial papilloma. All images are at 100× magnification. The scale bar for each image represents 250 μm.

Surgery was performed for the inverted papilloma in the nasal septum (Figure 3A). Tumor lesions were also observed in the right middle nasal turbinate (Figure 3B) and nasopharynx (Figure 3C) after tumor removal. The intraoperative diagnosis identified the lesion as a squamous epithelial papilloma. Surgery was concluded following excision of the lesion in the nasal septum considering the possibility of multiple lesions. Subsequent examination using flexible endoscopy revealed tumors in the right middle turbinate, nasopharynx, posterior wall of the oropharynx (Figure 3D), and left aryepiglottic fold (Figure 3E). Surgery for each lesion was performed 4 months after the initial procedure. Specimens excised from the nasal septum and middle nasal turbinate revealed an inverted papilloma (Figure 4A,B), whereas others showed squamous epithelial papilloma (Figure 4C–E). All pathological images are at 100× magnification, and the scale bar at the bottom left of each figure represents 250 μm. No recurrence was observed 100 days after the second surgery (Figure 3F–J).

## 3 METHODS

The patient was provided with an explanation of the study, and written consent was obtained at the initial visit. This study was approved by the Ethics Committee of the Hamamatsu Medical University (approval number: 19‐222).

DNA extraction from tumor tissue, HPV detection, and genotyping were performed according to previously described methods.^3^, ^7^ Samples were collected during each surgery. Specifically, the nasal septum tumor sample was obtained during the first surgery, and tumor samples from other sites were obtained during the second surgery. DNA extraction was performed using the QIAamp DNA Mini Kit (Qiagen, Hilden, Germany) following the manufacturer's instructions. All DNA extractions were conducted using fresh tissue samples collected during the surgical interventions.

HPV detection was performed using the consensus primer GP5+ (5′‐TTTGTTACTGTGGTAGATACTAC‐3′) and GP6+ (5′‐GAAAAATAAACTGTAAATCATATTC‐3′). Polymerase chain reaction (PCR) cycling involved an initial denaturation at 95°C for 2 min, followed by 50 cycles at 95°C for 30 s, 58°C for 1 min, and 72°C for 30 s, with a final extension at 72°C for 5 min using GoTaq Master Mix (Promega, Madison, WI, USA).

Direct sequence analysis was conducted using the PCR reaction solution on the 3500xL Genetic Analyzer (Applied Biosystems, Waltham, MA, USA). A sequence search was conducted using BLAST (https://blast.ncbi.nlm.nih.gov/) to determine the HPV type. In this case, HPV type 11 was detected in all the samples.

Blood samples for cfDNA analysis were immediately collected before the second surgery and at 49 and 100 days postoperatively (Figure 2). Blood samples (16 mL) were collected in a Cell‐Free DNA Collection Tube (Roche, Basel, Switzerland). After blood collection, the tubes were incubated at 4°C and allowed to stand for 15 min. Subsequently, centrifugation was performed at 1500 × *g* for 15 min and at 4°C to collect the plasma. cfDNA was extracted from 8 mL of plasma using a QIAamp MinElute ccfDNA Kit (Qiagen, Hilden, Germany).

Analysis of the viral load of HPV type 11 and the physical status has been described previously.^1^ Quantitative PCR was performed using a StepOne real‐time PCR System (Applied Biosystems). The following primer nucleotide sequences were used: HPV11 E2 forward, 5′‐GACCGTCCACTAACAACACC‐3′ and HPV11 E2 reverse, 5′‐TCCTTCTTTGGTGCTTGTTGT‐3′; HPV11 E6 forward, 5′‐CAAGCCGTTGTGTGAAATAGAA‐3′ and HPV11 E6 reverse, 5′‐CCAGCAGTGTAAGCAACGAC‐3′; beta‐actin forward, 5′‐TGCCCTCATTTCCCTCTCAG −3′ and beta‐actin reverse, 5′‐CGTACAGGTCTTTGCGGATG‐3′. Quantitative PCR involved an initial denaturation at 95°C for 30 s, followed by 50 cycles at 95°C for 5 s and 60°C for 30 s using TB Green Premix Ex Taq II (TaKaRa Bio, Shiga, Japan).

The HPV type 11 E2 and E6 plasmids^7^ were gifts from Taro Ikegami (Department of Otorhinolaryngology, Head and Neck Surgery, Graduate School of Medicine, University of the Ryukyus, Japan). They were serially diluted and standard curves were constructed. An external standard curve created using serial dilutions (0.3, 3, 30, and 300 ng) of human genomic placental DNA (Sigma–Aldrich, Darmstadt, Germany) for beta‐actin was amplified as an internal control. The viral load was defined as the copy number of HPV E6 per 1 ng of DNA. The ratio of E2 to E6 copy numbers defines the viral physical status, with E2/E6> 1 indicating episomal dominance, 0 <E2/E6 < 1 indicating mixed episomal type and integrated type, and E2/E6= 0 indicating integration.^8^, ^9^

Statistical analysis was performed using SPSS v.26 (IBM, Armonk, NY, USA). We calculated the variance and standard deviation for the viral load and E2/E6 values.

## 4 CONCLUSIONS AND RESULTS

The viral load of each tumor was as follows: nasal septum 2.32 × 10^6^ copies/ng DNA, middle turbinate 7.20 × 10^5^ copies/ng DNA, nasopharynx 3.46 × 10^6^ copies/ng DNA, oropharynx 4.56 × 10^5^ copies/ng DNA, and aryepiglottic fold 2.11 × 10^4^ copies/ng DNA. Conversely, the E2/E6 ratios, indicating the integration rate of HPV into the host, were as follows: nasal septum 0.43, middle turbinate 0.41, nasopharynx 0.38, oropharynx 0.38, and aryepiglottic fold 0.39 (Table 1). Lower E2/E6 values indicate a higher degree of HPV integration into the host. When HPV integrates, it may lead to carcinogenesis and is considered an indicator of disease severity, as in cervical cancer.^10^ In this study, as in previous studies,^3^, ^11^ the E2/E6 values were approximately 0.4, indicating a mixed type. The variance of the viral load measured from tumor tissues was 2.08 × 10^12^ and the standard deviation was 1.44 × 10^6^ Meanwhile, the variance of the E2/E6 ratio was <0.01, and the standard deviation was 0.02. These results indicate that while the viral load varies among the multiple tumors in the same patient, the ratio of HPV integration into the tumor tissue remains consistent.

**TABLE 1:** Human papillomavirus viral load and physical status.

	Viral load (copies/ng DNA)	E2/E6	HPV status
Nasal septum	2.32 × 10^6^	0.43	Mix
Middle turbinate	7.20 × 10^5^	0.41	Mix
Nasopharynx	3.46 × 10^6^	0.38	Mix
Oropharynx	4.56 × 10^5^	0.38	Mix
Aryepiglottic fold	2.11 × 10^4^	0.39	Mix
Liquid biopsy			
1	3.33 × 10^3^	‐	‐
2	ND	‐	‐
3	ND	‐	‐

Abbreviations: ND, not detected; HPV, human papillomavirus.

**FIGURE 5:**
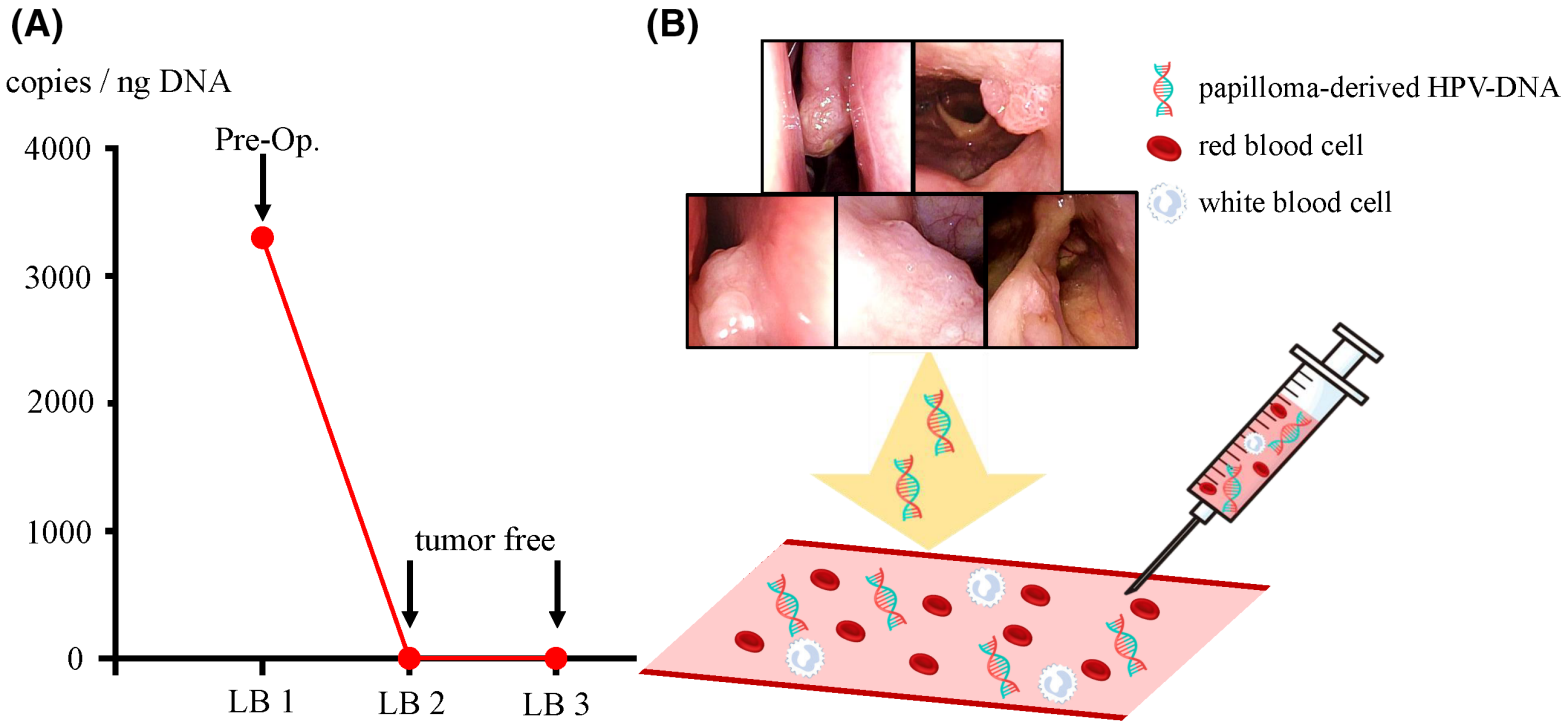
(A) Charting the evolution of the human papillomavirus (HPV) viral load in cell‐free DNA (cfDNA). While viral load was detectable preoperatively, it became undetectable postoperatively, aligning with clinical observations of achieving remission. LB, liquid biopsy. (B) Feasibility of measuring viral load of circulating HPV‐DNA derived from papillomas present in the bloodstream. Pre‐Op, preoperative.

cfDNA was detected in the sample just before surgery, with a viral load of 3.33 × 10^3^ copies/ng DNA. Postoperatively, the viral load could not be measured, which aligned with the clinical course of remission (Figure 5A).

It is possible to detect papilloma‐derived HPV‐DNA and measure the HPV viral load in cfDNA from the blood of patients with RRP (Figure 5B). This viral load was undetectable during remission, suggesting its potential as a novel biomarker.

## 5 DISCUSSION

cfDNA analysis is a minimally invasive method that enables real‐time monitoring of minimal residual disease, treatment efficacy, and cancer recurrence.^12^ In HPV‐associated oropharyngeal cancer, HPV viral load^5^, ^13^, ^14^ and methylation markers^15^ in cfDNA have been reported to be associated with disease status. The analysis of cfDNA in benign tumors (including RRP) has not been previously reported. Our study demonstrated the feasibility of cfDNA analysis in patients with RRP using the viral load as an indicator. Additionally, the viral load of the cfDNA became undetectable postoperatively, which correlates with clinical remission. RRP varies in severity, with some cases achieving remission after a few surgeries while others require lifelong surgical intervention.^1^, ^2^, ^3^, ^16^ The Derkay Score^4^ is a well‐known tool for assessing the severity of RRP, but evaluation based on HPV viral load also holds potential as a convenient method for evaluating disease severity.

In our case, distinct regions of squamous epithelial papilloma and inverted papilloma were observed depending on the site of tumor presence; however, determination of viral load measurements was feasible from all tumors. Therefore, it is considered that all tumors were associated with HPV type 11. Inverted papilloma may occur in the nasal cavity during RRP, suggesting the possibility of differing pathological expressions based on the site.^17^ Additionally, the viral load significantly varied among the different tumors. While precise size measurement is challenging, lesions in the nasal septum and nasopharynx were notably larger than other lesions in this case. We showed that the copy number of HPV E6, a reported factor contributing to carcinogenesis,^18^, ^19^, ^20^ may correlate with tumor size.

Inverted papilloma of the nasal cavity and RRP coexist with HPV in episomal and integrated states.^11^, ^21^ The status of HPV within host cells is not strictly defined, but is simply represented by the ratio of HPV‐E2 to HPV‐E6. The E2/E6 values did not show statistical differences among the individual tumors and were comparable to our previous report.^3^ This suggests that each tumor was seeded by a single tumor.

The limitations of this study were that it consisted of only a single case. Additionally, this case did not present a recurrence; thus, it could not be determined whether the measurement of HPV viral load in the cfDNA can reliably detect tumor recurrence. Moreover, real‐time PCR may have resulted in lower sensitivity for cfDNA detection compared with digital PCR. Nonetheless, the crucial observation that the viral load of cfDNA becomes undetectable after surgery highlights the potential of the cfDNA viral load as a novel biomarker for RRP.

This study revealed the detectability of cfDNA in RRP. Similar to cancer research, tumor‐derived genetic markers in the blood may enable to predict the efficacy of drug treatments. Systemic administration of bevacizumab for RRP has been reported to be highly effective and is gaining attention.^1^, ^22^ HPV viral load in the cfDNA of RRP patients may potentially be used to assess the efficacy of systemic bevacizumab therapy. Therefore, measuring HPV viral load of the cfDNA for RRP can potentially reflect various treatment effects such as surgery, HPV vaccination, and bevacizumab administration in real time. Furthermore, disseminated lesions to lower airways such as the lungs may present challenges in the differential diagnosis with other conditions solely based on imaging findings such as CT scans. Measuring viral load of the cfDNA could be effective in distinguishing lower airway lesions. Recently, there has been significant progress in the development of artificial intelligence‐based diagnostic support technologies.^23^ Thus, in the future, it might be possible to create artificial intelligence diagnostic tools that predict the severity of RRP and its malignant transformation using factors such as HPV viral load in cfDNA, pathological findings, and clinical data. This could be achieved through large‐scale studies.

## AUTHOR CONTRIBUTIONS

**Satoshi Yamada:** Conceptualization; data curation; formal analysis; investigation; methodology; project administration; resources; supervision; visualization; writing – original draft; writing – review and editing. **Kotaro Kano:** Data curation; investigation; visualization; writing – original draft; writing – review and editing. **Ryuji Ishikawa:** Data curation; visualization; writing – original draft; writing – review and editing. **Atsushi Imai:** Data curation; funding acquisition; investigation; writing – original draft; writing – review and editing. **Daiki Mochizuki:** Data curation; formal analysis; funding acquisition; investigation; writing – original draft; writing – review and editing. **Kotaro Morita:** Data curation; writing – original draft; writing – review and editing. **Kazutaka Takeuchi:** Data curation; formal analysis; investigation; methodology; writing – original draft; writing – review and editing. **Yoshinori Takizawa:** Data curation; visualization; writing – original draft; writing – review and editing. **Hideya Kawasaki:** Data curation; funding acquisition; validation; visualization; writing – original draft; writing – review and editing. **Kiyoshi Misawa:** Conceptualization; data curation; funding acquisition; validation; writing – original draft; writing – review and editing.

## FUNDING INFORMATION

This study was funded by a Grant‐in‐Aid for Scientific Research (Nos. 23H03054, 23K08960, 23K08981, and 23K08911) from the Ministry of Education, Culture, Sports, Science, and Technology of Japan.

## CONFLICT OF INTEREST STATEMENT

The authors declare no competing interests.

## ETHICS STATEMENT

This study was approved by the Ethics Committee of Hamamatsu Medical University (approval number: 19‐222).

## CONSENT

Written informed consent was obtained from the patient to publish this report in accordance with the journal's patient consent policy.

## Data Availability

All data in this article are presented in the manuscript, figures, and table.
